# Impact of post-dialysis calcium level on *ex vivo* rat aortic wall calcification

**DOI:** 10.1371/journal.pone.0183730

**Published:** 2017-08-23

**Authors:** Daniel Azpiazu, Emilio González-Parra, Alberto Ortiz, Jesús Egido, Ricardo Villa-Bellosta

**Affiliations:** Fundación Instituto de Investigación Sanitaria, Fundación Jiménez Díaz (FIIS-FJD), Madrid, Spain; University Medical Center Utrecht, NETHERLANDS

## Abstract

**Objectives:**

Vascular calcification is a frequent complication in chronic haemodialysis patients and is associated with adverse outcomes. Serum calcium and phosphate levels and imbalances in calcification regulators are thought to contribute to the process. In this regard, the dialysate calcium concentration is a modifiable tool for modulating the risk of vascular calcification. We explored pre- and post-dialysis phosphate and calcium concentrations in stable chronic haemodialysis patients treated by dialysis with the KDIGO-suggested 1.5 mmol/L calcium dialysate to investigate the effects on *ex vivo* calcification of rat aortic rings.

**Approach and results:**

At the end of haemodialysis, mean serum calcium levels were increased in 88% of paired pre-/post-dialysis samples, while mean serum phosphate and parathyroid hormone levels were decreased. Rat aortic ring cultures grown at the same calcium and phosphate concentrations revealed that pre- and post-dialysis resulted in a similar degree of calcification. By contrast, haemodialysis with unchanged serum calcium resulted in a 5-fold reduction in calcium deposition.

**Conclusion:**

Dialysis with the widely prescribed 1.5 mmol/L calcium dose results in persistent high serum calcification potential in a sizable proportion of patients, driven by increased post-dialysis calcium concentration. This could potentially be mitigated by individualising dialysate calcium dosage based on pre-dialysis serum calcium levels.

## Introduction

Ectopic calcification of blood vessels, also known as vascular calcification, is among the most common complications in patients receiving chronic haemodialysis, and is associated with cardiovascular events and all-cause mortality [1,2]. Elevated serum phosphate is a predictable feature of chronic haemodialysis patients in the absence of dietary phosphate restrictions or supplemental phosphate binders, and contributes to the substantial morbidity and mortality rates in this population [2–4]. Accumulation of calcium-phosphate crystals in the aortic wall is the main hallmark of vascular calcification. Incremental increases in the phosphate concentration increase calcium-phosphate crystal formation [5] by shifting the H_2_PO_4_^-^/HPO_4_^2-^ equilibrium to the right [6], which promotes the association of HPO_4_^2-^ with calcium to form brushite (CaHPO_4_2H_2_O), octacalcium phosphate (Ca_8_H_2_(PO_4_)_6_5H_2_O) and hydroxyapatite (Ca_10_(PO_4_)_6_(OH)), the main crystal forms in ectopic calcification and bone.

The calcium-phosphate product (CaxPi) is also associated with an increased risk of vascular calcification; however, the mortality risk associated with CaxPi levels is similar to that of phosphate alone [3,4], and this product is not a determinant of vascular calcification [5,7]. The calcium concentration is a greater determinant of calcium-phosphate crystal formation than the phosphate concentration [5]. For example, calcification may not be induced by elevated phosphate concentration when the calcium concentration is low [5]. Therefore, even if phosphate is eliminated during dialysis, calcification can still occur provided that the calcium concentration is increased.

Since the dialysate calcium concentration is a modifiable factor that determines whether serum calcium levels increase during haemodialysis, in the present study we analysed the effects of calcium and phosphate concentrations during haemodialysis on the deposition of calcium-phosphate crystals in the rat aortic wall.

## Materials and methods

### Haemodialysis conditions and sampling

All patients (from University Hospital Fundación Jiménez Díaz, n = 68) received a conventional purely diffusive 4 h (mid-week) haemodialysis session without haemodiafiltration using a high-flux helixone dialyser (Fresenius; CUF, 59 ml/h/mmHg; surface, 1.8 m^2^). The dialysate composition was 1.5 mmol/L calcium, 35 mmol/L bicarbonate, 0.75 mmol/L potassium, 0.5 mmol/L magnesium and 140 mmol/L sodium. Thus, the dialysate calcium concentration was within the lower range of the Kidney Disease: Improving Global Outcomes (KDIGO) Chronic Kidney Disease-Mineral and Bone Disorder (CKD-MBD) guidelines of 1.25–1.50 mmol/L [8]. The main clinical characteristics of patients are summarised in Table 1.

**Table 1:** Demographic and clinical characteristics of the study population. Data expressed as mean±SD or %.

Age (years)	65.3 ± 14.9
Dry weight (Kg)	66.1 ± 12.2
Dialysis vintage (years)	4.3 ± 4.3
Males (%)	51.5
Kt / v urea	1.47 ± 0.3
*Vitamin D therapy (%)	55.9
Cinacalcet (%)	19.1
Calcium-based phosphate binders (%)	42.6
Sevelamer (%)	36.7

*25OH vitamin D, calcitriol or paricalcitol.

Pre- and post-haemodialysis blood samples were collected in heparin-containing tubes and immediately centrifuged at 4°C for 5 min at 5000 rpm. Plasma samples were frozen in liquid nitrogen and stored at -80°C until further use. In 24 patients, phosphate levels were measured in two independent extractions at each time point. This study was conducted according to the Declaration of Helsinki and approved by the Ethics Committee of Research of University Hospital Fundación Jiménez Díaz. Participants were identified by number only. Inclusion criteria stipulated that only consenting stable adults on chronic haemodialysis with a life expectancy of over 6 months were included. There were no excluding criteria based on compliance, calcium, phosphate, parathyroid hormone (PTH) or vitamin D levels, or concomitant medications. Patients with positive serology for human immunodeficiency virus (HIV), hepatitis B surface antigen (HBsAg) or anti-hepatitis virus (HVC) antibodies or other known active infection were excluded.

### Animals

Male Sprague-Dawley rats (body weight, 200–400 g) were obtained from Charles River Laboratories (Barcelona, Spain). The protocol was approved by the Fundación Jiménez Díaz (FIIS-FJD) ethics committee and conformed to directive 2010/63EU and recommendation 2007/526/EC regarding the protection of animals used for experimental and other scientific purposes, enforced in Spanish law under RD1201/2005.

### Aorta isolation and calcification assay

Rats were euthanised by carbon dioxide inhalation, and aortas were perfused with saline and removed according to previous descriptions [9,10] and frozen immediately. Four or five rings were obtained from each aorta (n = 24) and cultured *ex vivo* (37°C, in a humidified atmosphere of 5% CO_2_) in 6-well culture plates using Minimum Essential Medium (MEM; Gibco, Paisley, UK) containing 1 mM L-glutamine, 100 IU/mL penicillin, 100 μg/mL streptomycin and the indicated calcium and phosphate concentration, as previously described [5]. The medium was replaced every 2 days and contained ^45^calcium (Perkin Elmer; Boston, USA) as a radiotracer. After 6 days of incubation, aortic rings were dried and weighed, and radioactivity was measured using liquid scintillation (Ultima Gold, 6013329, Perkin Elmer) and a liquid scintillation counter (Tri-Carb 2900TR, Perkin Elmer).

### Biochemical assessments

Calcium, ionized calcium and intact PTH levels in plasma were quantified by routine clinical biochemistry laboratory methods. For phosphate quantification, the Phosphate Assay Kit (MAK030-1KT, Sigma-Aldrich) was used according to the manufacturer’s instructions.

### Statistical analyses

**Fig 1:**
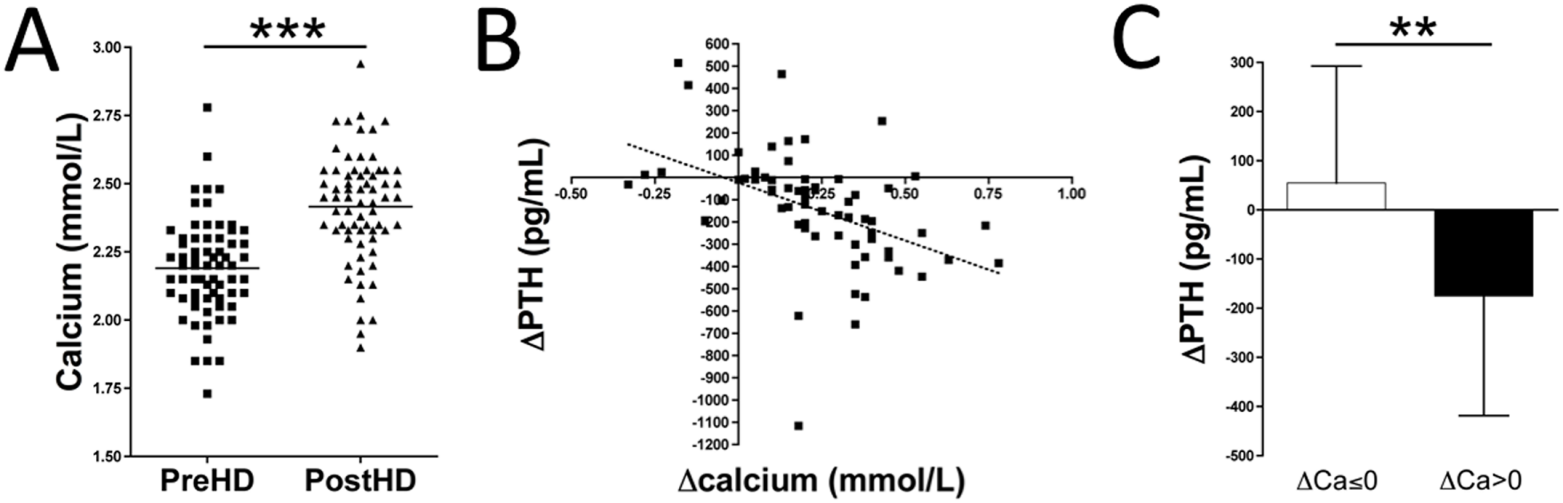
Calcium and PTH concentration in haemodialysis patients. (**A**) Plasma calcium concentration in pre- and post-haemodialysis samples (n = 68). The Wilcoxon matched pairs test was used for statistical analysis. (**B**) Scatter graph displaying a significant (*p* < 0.001) correlation between ΔCalcium (ΔCa, the difference between post- and pre-haemodialysis calcium concentration) and ΔPTH (the difference between post- and pre-haemodialysis PTH concentration). (**C**) ΔPTH based on ΔCalcium (ΔCa ≤0 or ΔCa >0 mmol/L). The Mann-Whitney test was used for statistical analysis. **p < 0.01; ***p < 0.001. **PreHD**, pre-haemodialysis; **PostHD**, post-haemodialysis.

**Fig 2:**
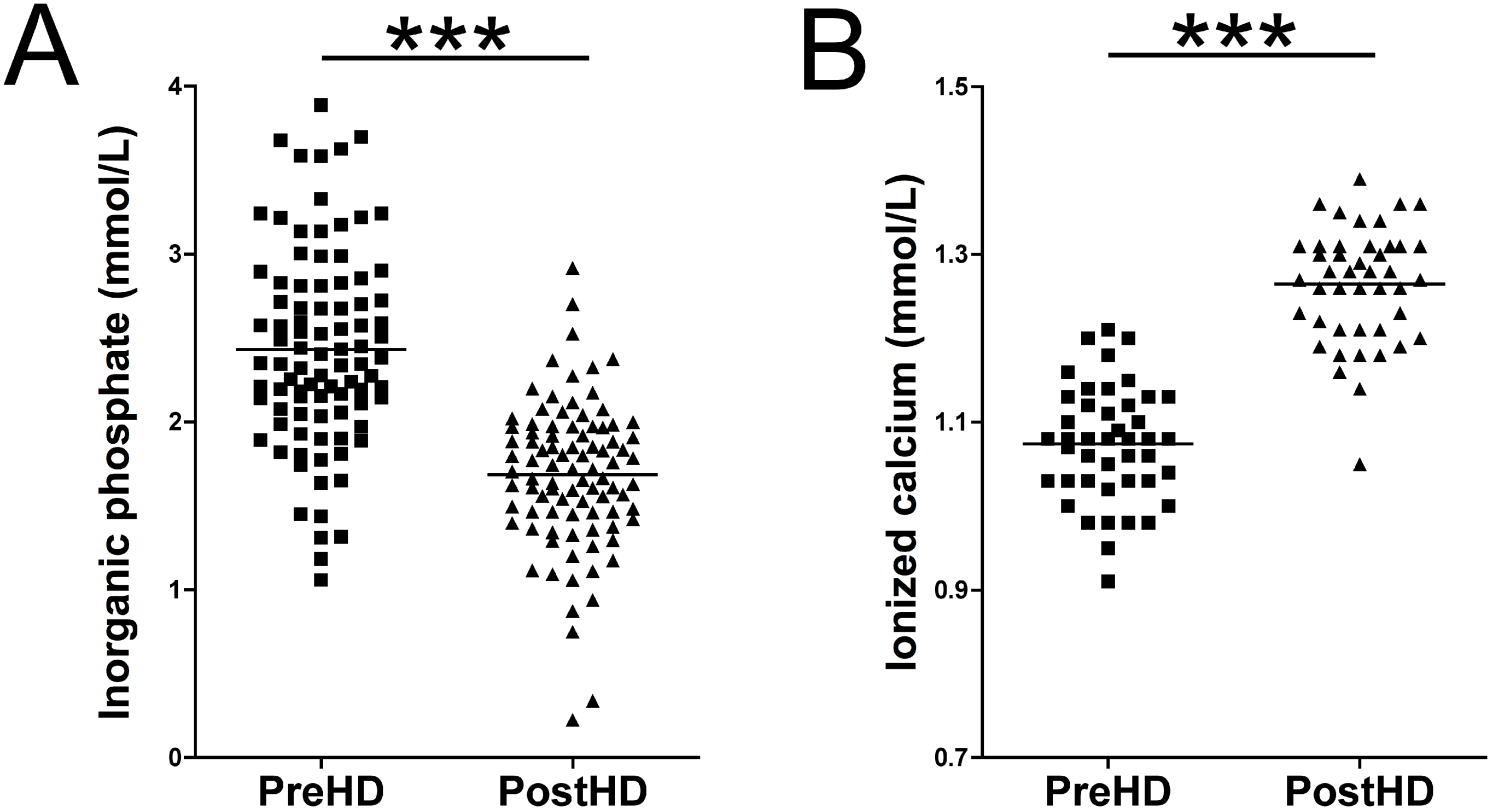
Inorganic phosphate and ionized calcium concentration in haemodialysis patients. (**A**) Plasma phosphate concentration in pre- and post-haemodialysis samples (n = 92). (**B**) Plasma ionized calcium concentration in pre- and post-haemodialysis samples (n = 45). The Wilcoxon matched pairs test (pre- versus post-haemodialysis) was used for statistical analysis. ***p < 0.001. **PreHD**, pre-haemodialysis; **PostHD**, post-haemodialysis.

**Fig 3:**
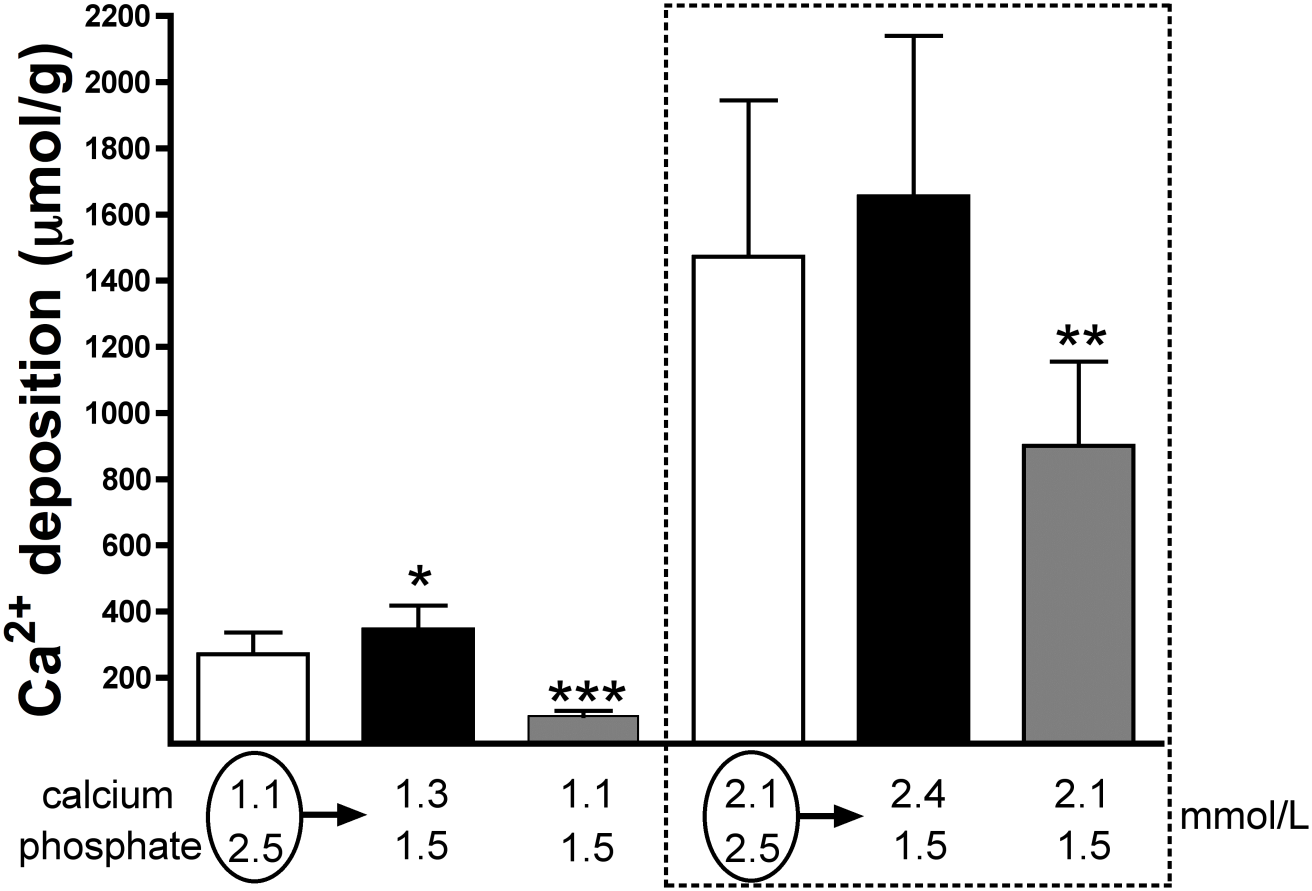
Calcium deposition in the rat aortic wall *ex vivo* under calcium and phosphate concentrations matching pre- and post-haemodialysis levels in haemodialysis patients. Rat aortic rings were incubated *ex vivo* in MEM medium containing the indicated concentrations of calcium and phosphate. The medium was replaced every 2 days and contained ^45^calcium as a radiotracer. After 6 days of incubation, aortic rings were dried and radioactivity was measured by liquid scintillation counting. Results are represented as mean ± SD from three independent experiments, with 15 or 16 rings per condition (five or six rings per condition per experiment). One-way ANOVA and Tukey’s multicomparison test were used for statistical analysis. Calcium and phosphate concentrations are in mmol/L. *p < 0.05; ***p < 0.001. Pre-dialysis conditions were used as a reference. **PreHD**, pre-haemodialysis; **PostHD**, post-haemodialysis.

Results in Figs 1, 2, 3 and Table 1 are presented as mean and standard deviation (SD) or median (interquartile range). The Wilcoxon matched pairs test was used for statistical analysis of results presented in Figs 1A and 2. The Mann-Whitney test was used in Fig 1C. One-way ANOVA and Tukey’s multicomparison test were used in Fig 3. Statistical significance was determined with GraphPad Prism 5 and was assigned at *p* < 0.05.

## Results

Plasma calcium levels (n = 68) were significantly increased by 14.3% (*p* < 0.001) post-haemodialysis (2.4 ± 0.2 mmol/L) compared with pre-haemodialysis (2.1 ± 0.2 mmol/L) values. Serum calcium was increased in 88.2% (60/68) of the paired pre-/post-dialysis samples analysed (Fig 1A, S1 Table). Changes in the PTH level during haemodialysis were correlated with changes in calcium levels. Fig 1B shows a significant correlation (R^2^ = 0.2; *p* < 0.001) between post- and pre-haemodialysis calcium level differences (ΔCalcium) and post- and pre-haemodialysis PTH level differences (ΔPTH). Thus, PTH levels were increased during haemodialysis (median (IQR) = 1 (-102.1; 113.7) pg/mL; n = 10) when ΔCalcium was ≤0 mmol/L; (Fig 1C ΔCa≤0). By contrast, PTH levels were significantly decreased (*p* = 0.007; median (IQR) = -160.2 (-300.1; -43.6) pg/mL; n = 58) when ΔCalcium was >0 mmol/L (Fig 1C ΔCa>0).

Plasma phosphate levels were significantly decreased by 32.0% (*p* < 0.001) post-haemodialysis versus pre-haemodialysis (2.5 ± 0.6 vs. 1.7 ± 0.4 mmol/L, respectively; Fig 2A). Pre-haemodialysis phosphate levels ranged from 1.05 to 3.89 mmol/L, and in 93.5% of cases they were in the hyperphosphatemia range (>1.5 mmol/L phosphate). A decrease in the phosphate level post-haemodialysis was observed in all paired samples analysed (n = 92). Interestingly, 69.6% of post-haemodialysis plasma phosphate samples remained above 1.5 mmol/L phosphate, and 52.2% were above 1.7 mmol/L phosphate. In addition to plasma calcium levels, ionized calcium levels (Fig 2B) were also significantly increased (*p* < 0.001) in post-haemodialysis plasma (1.26 ± 0.09—mmol/L) versus pre-haemodialysis plasma (1.07 ± 0.09—mmol/L).

Finally, aortic rings were cultured in medium containing the same post-haemodialysis Ca and Pi concentrations (2.4 mmol/L and 1.5 mmol/L, respectively) or pre-haemodialysis Ca and Pi concentrations (2.1 mmol/L and 2.5 mmol/L, respectively) as those measured above, and the results are shown in Fig 3 and S2 Table. No significant differences were observed in calcium deposition on the aortic wall *ex vivo* (1654.5 ± 485.2 μmol/g and 1472.9 ± 472.1 μmol/g, respectively; n = 15) between Ca and Pi concentrations measured pre- and post-dialysis. By contrast, the amount of calcium deposited in the aortic wall was significantly lower (900.6 ± 254.5 μmol/g; *p* < 0.01) when the calcium concentration was 2.1 mmol/L (as would be expected in post-haemodialysis samples if the calcium level did not change during dialysis) and the phosphate concentration was 1.5 mmol/L (as observed in post-haemodialysis samples).

In addition, similar results were obtained when the ionized calcium concentration measured above (1.1 mmol/L and 1.3 mmol/L pre- and post-haemodialysis, respectively) was used. In this case, a significant increase (*p* < 0.05; n = 16) in calcium deposited on the aortic wall was observed under post-haemodialysis conditions (345.8 ± 72.3 μmol/g; 1.3 mmol/L calcium and 1.5 mmol/L phosphate) compared with pre-dialysis conditions (270.5 ± 66.4 μmol/g; 1.1 mmol/L calcium and 2.5 mmol/L phosphate). By contrast, the amount of calcium deposited in the aortic wall was significantly lower (83.9 ± 16.1 μmol/g; *p* < 0.001) when the calcium concentration was 1.1 mmol/L and the phosphate concentration was 1.5 mmol/L (Fig 3).

## Discussion

In biological systems, phosphorus is found in hard tissues (mainly bone, 85%), soft tissues (14%) and extracellular fluid (1%). Of the 1% that is present in extracellular fluid, 85% exists as the free inorganic phosphate ion (Pi) in solution. The normal range for plasma inorganic phosphate is 0.8–1.5 mmol/L. An elevated phosphate level is a risk factor for vascular calcification, cardiovascular disease and mortality [3,4]; therefore, phosphate is considered a uremic toxin that needs to be eliminated during haemodialysis. In this study, we observed a reduction in the plasma phosphate concentration during haemodialysis, from 2.5 mmol/L (pre-dialysis) to 1.7 mmol/L (post-dialysis). However, ~70% of post-haemodialysis samples remained above 1.5 mmol/L. By contrast, the calcium concentration was higher in post-dialysis plasma in 88% of paired samples analysed (2.1 vs. 2.4 mmol/L for mean pre- and post-dialysis plasma calcium concentrations, respectively). Under these pre- and post-haemodialysis levels of calcium and phosphate, calcium accumulation in the aortic wall was similar. Thus, a conventional haemodialysis session, using a fixed and non-individualised dialysate calcium dosage within the range suggested by international guidelines [8] did not improve the vascular calcification risk associated with calcium and phosphate concentrations found in serum. By contrast, a significant reduction in calcium accumulation in the rat aortic wall was observed when mimicking post-haemodialysis conditions associated with neutral post-dialysis calcium levels. This assumes that post-haemodialysis calcium remained unchanged during the haemodialysis session (2.1 mmol/L calcium and 1.5 mmol/L phosphate). Similar results were also observed when ionized calcium levels were used.

Post-dialysis calcium levels depend on pre-dialysis plasma calcium, dialysate calcium and ultrafiltration. Lower post-dialysis ionised calcium levels are associated with an increase in serum PTH levels, while an increase in post-dialysis ionised calcium results in suppression of PTH, as observed both in the present study and previously [11,12]. While we did not perform detailed ionised calcium balance studies, the observed suppression of serum PTH in patients in whom serum calcium increased during dialysis suggests that the observed increase in serum calcium was physiologically relevant. In this regard, increased calcium levels are associated with the maintenance of a similar procalcific effect *ex vivo*, despite the reduction in the phosphate concentration, as shown here. Thus, the present findings support the concept of personalisation of dialysate calcium based on individual patient pre-dialysis serum calcium to maintain balanced calcium levels. The current KDIGO CKD-MBD guidelines also emphasise the concept of individualisation of dialysate calcium [8]. Our findings suggest that dialysate calcium of 1.5 mmol/L results in a persistent post-dialysis risk of vascular calcification in a sizable proportion of patients, and further individualisation would be desirable. Moreover, Ok et al, observed a reduction in the progression of coronary artery calcification when the dialysate calcium level was 1.25 mmol/L, supporting the notion that lowering dialysate calcium would be effective in preventing progression of vascular calcification [13]. However, if the dialysate calcium concentration is too low, this could result in haemodynamic instability and arrhythmia, hence the need for dialysate calcium individualisation.

Among the weaknesses of the present study, in the rat aortic ring culture conditions, we did not model the increase in pH and serum bicarbonate associated with the haemodialysis procedure, which could affect the positive base balance and cause metabolic alkalosis [14,15]. Given that metabolic alkalosis, or even the correction of acidosis, is associated with increased vascular calcification risk under uremic conditions [16–19], our approach may have underestimated the procalcification effect of post-haemodialysis calcium and phosphate concentrations. For example, Villa-Bellosta et al. observed an increase in calcium-phosphate deposition at alkaline pH in an *in vitro* calcification model, while no calcium-phosphate deposition was observed under acidosis conditions [5]. Moreover, post-dialysis alkalinisation results in increased alkaline phosphatase activity [20]. This consequently lowers the availability of pyrophosphate, the main endogenous inhibitor of calcium-phosphate crystal formation and growth. Finally, our experiments did not model the dynamic nature of the calcium concentration during the 48 h between haemodialysis sessions. However, haemodialysis patients may experience repeated (thrice weekly) serum calcium peaks over many years (decades in countries with low transplantation rates), which, combined with evidence of positive calcium balance in a significant number of patients during haemodialysis [21], supports the clinical relevance of the findings.

In conclusion, the present findings suggest that the frequent current practice of using a one-size-fits-all dialysate calcium concentration may result in the persistence of calcification-prone serum calcium and phosphate concentrations post-dialysis, and support the concept of dialysate calcium concentration individualisation. While the international guidelines already propose the individualisation of the dialysate calcium concentration, the suggested default calcium dialysate value may still be too high and might promote vascular calcification in a sizable proportion of patients [8].

## Supporting information

S1 Table Experimental results Fig 1. (PDF)

S2 Table Experimental results Fig 3. (PDF)
