# Supplementary material for: Impact of post-dialysis calcium level on ex vivo rat aortic wall calcification
Source: PLoS One. 2017 Aug 23;12(8):e0183730. doi: 10.1371/journal.pone.0183730 (PMC5568142; doi:10.1371/journal.pone.0183730)
Supplement: S1 Table — (PDF) [file pone.0183730.s001.pdf]

S1 Table Results Fig 1

Impact of post-dialysis calcium levels on ex vivo rat aortic wall calcification

Daniel Azpiazu, Emilio González-Parra, Alberto Ortiz, Jesús Egido, Ricardo Villa-Bellosta.

Patient	Calcium PreHD (mmol/L)	Calcium PostHD (mmol/L)	ΔCalcium PostHD-PreHD	PTH PreHD (pg/mL)	PTH PostHD (pg/mL)	ΔPTH PostHD-PreHD
1	2,23	1,90	-0,33	88,3	56,9	-31,4
2	2,78	2,50	-0,28	321,1	333,1	12,0
3	2,23	2,00	-0,23	87,8	111,8	24,0
4	2,60	2,43	-0,18	622,0	1136,9	514,9
5	2,35	2,20	-0,15	97,9	512,9	415,0
6	2,23	2,13	-0,10	443,6	246,7	-196,9
7	2,10	2,00	-0,10	565,2	373,1	-192,1
8	2,23	2,18	-0,05	259,6	157,5	-102,1
9	2,33	2,33	0,00	17,4	7,4	-10,0
10	2,15	2,15	0,00	501,5	615,2	113,7
11	2,48	2,50	0,02	19,5	14,2	-5,3
12	2,08	2,13	0,05	443,2	469,6	26,4
13	2,43	2,48	0,05	110,1	100,5	-9,6
14	2,28	2,35	0,08	139,8	139,8	0,0
15	1,85	1,95	0,10	78,3	217,7	139,4
16	2,25	2,35	0,10	81,2	16,8	-64,4
17	2,15	2,25	0,10	226,1	171,7	-54,4
18	1,98	2,08	0,10	189,6	178,0	-11,6
19	2,48	2,60	0,13	155,1	17,6	-137,5
20	2,48	2,60	0,13	549,7	1014,5	464,8
21	2,13	2,28	0,15	88,7	40,7	-48,0
22	2,30	2,45	0,15	173,6	41,4	-132,2
23	2,05	2,20	0,15	201,6	275,1	73,5
24	2,30	2,45	0,15	339,0	502,7	163,7
25	2,20	2,38	0,18	965,8	344,5	-621,3
26	2,23	2,40	0,18	251,7	40,9	-210,8
27	2,15	2,33	0,18	1665,9	550,2	-1115,7
28	2,15	2,33	0,18	94,1	33,4	-60,7
29	2,35	2,55	0,20	348,5	120,8	-227,7
30	2,20	2,40	0,20	166,3	45,5	-120,8
31	2,10	2,30	0,20	472,8	644,6	171,8
32	2,15	2,35	0,20	352,1	148,0	-204,1
33	2,25	2,45	0,20	108,0	22,9	-85,1
34	2,15	2,35	0,20	76,7	19,9	-56,8
35	2,30	2,50	0,20	15,0	7,4	-7,6
36	2,33	2,55	0,23	90,6	33,5	-57,1
37	2,30	2,53	0,23	424,1	160,2	-263,9
38	2,00	2,23	0,23	142,3	98,7	-43,6
39	2,28	2,53	0,25	443,3	292,3	-151,0
40	2,23	2,53	0,30	366,0	105,3	-260,7
41	2,20	2,50	0,30	200,1	30,3	-169,8

**42**	2,05	2,35	0,30	473,5	304,1	-169,4
**43**	2,43	2,73	0,30	113,3	106,3	-7,0
**44**	2,23	2,55	0,33	139,6	31,0	-108,6
**45**	2,15	2,48	0,33	280,5	101,6	-178,9
**46**	2,20	2,55	0,35	425,1	122,9	-302,2
**47**	2,08	2,43	0,35	353,6	53,5	-300,1
**48**	2,25	2,60	0,35	977,5	317,7	-659,8
**49**	2,00	2,35	0,35	1244,7	721,5	-523,2
**50**	1,98	2,33	0,35	468,6	76,3	-392,3
**51**	2,13	2,48	0,35	143,8	65,6	-78,2
**52**	2,03	2,40	0,38	745,7	208,9	-536,8
**53**	2,35	2,73	0,38	562,4	205,1	-357,3
**54**	2,10	2,48	0,38	246,5	60,2	-186,3
**55**	2,33	2,73	0,40	609,3	333,5	-275,8
**56**	2,35	2,75	0,40	298,9	102,6	-196,3
**57**	2,05	2,45	0,40	396,3	148,6	-247,7
**58**	2,28	2,70	0,43	379,3	633,4	254,1
**59**	2,10	2,55	0,45	686,0	327,1	-358,9
**60**	1,85	2,30	0,45	474,0	143,2	-330,8
**61**	2,10	2,55	0,45	110,0	61,4	-48,6
**62**	1,85	2,33	0,48	662,2	243,5	-418,7
**63**	1,93	2,45	0,53	364,3	370,1	5,8
**64**	2,08	2,63	0,55	563,5	118,4	-445,1
**65**	2,00	2,55	0,55	328,0	78,7	-249,3
**66**	2,08	2,70	0,63	521,7	151,9	-369,8
**67**	2,20	2,94	0,74	377,0	161,0	-216,0
**68**	1,73	2,50	0,78	547,9	163,8	-384,1
