# Supplementary material for: Impact of post-dialysis calcium level on ex vivo rat aortic wall calcification
Source: PLoS One. 2017 Aug 23;12(8):e0183730. doi: 10.1371/journal.pone.0183730 (PMC5568142; doi:10.1371/journal.pone.0183730)
Supplement: S2 Table — (PDF) [file pone.0183730.s002.pdf]

S2 Table **Calcium deposition**

Impact of post-dialysis calcium levels on ex vivo rat aortic wall calcification

Daniel Azpiazu, Emilio González-Parra, Alberto Ortiz, Jesús Egido, Ricardo Villa-Bellosta.

1.1	1.3	1.1	2.1	2.4	2.1	Ca (mmol/L)
2.5	1.5	1.5	2.5	1.5	1.5	Pi (mmol/L)
263,659	331,812	99,732	1606,790	1108,570	1393,662	
232,378	325,082	109,613	1641,835	1122,695	1432,180	
307,987	344,009	80,712	1994,944	1816,155	825,329	
168,487	299,155	75,010	910,111	1414,365	776,695	
360,131	331,400	72,220	955,937	1441,276	767,197	Experiment 1
229,143	310,021	70,092	909,542	1422,567	882,078	
352,954	517,609	62,279	884,852	1393,043	700,846	
218,299	270,650	96,856	966,513	1460,848	780,964	
270,046	341,343	102,776	1977,697	1785,563	811,226	
237,914	333,857	109,585	1877,213	2493,166	681,843	Experiment 2
322,863	350,958	81,508	1887,379	2489,085	779,881	
175,494	314,537	76,559	1872,637	2469,965	760,245	
368,078	336,324	72,910	1640,925	1121,937	1309,291	
233,930	317,742	72,338	973,679	1465,267	880,437	
359,494	526,982	63,096	1993,249	1812,968	726,788	Experiment 3
226,522	281,942	97,482				
